# Small molecule natural compound agonist of SIRT3 as a therapeutic target for the treatment of intervertebral disc degeneration

**DOI:** 10.1038/s12276-018-0173-3

**Published:** 2018-11-12

**Authors:** Jianle Wang, Majid Nisar, Chongan Huang, Xiangxiang Pan, Dongdong Lin, Gang Zheng, Haiming Jin, Deheng Chen, Naifeng Tian, Qianyu Huang, Yue Duan, Yingzhao Yan, Ke Wang, Congcong Wu, Jianing Hu, Xiaolei Zhang, Xiangyang Wang

**Affiliations:** 1 Department of Orthopaedic Surgery, The Second Affiliated Hospital and Yuying Children’s Hospital of Wenzhou Medical University, Wenzhou,, 325027 Zhejiang China; 2 Zhejiang Provincial Key Laboratory of Orthopaedics, Wenzhou,, 325027 Zhejiang China; 3 Department of Neurosurgery Surgery, The Second Affiliated Hospital and Yuying Children’s Hospital of Wenzhou Medical University, Wenzhou,, 325027 Zhejiang China; 4 Department of Anesthesiology and Operating Room, The Second Affiliated Hospital and Yuying Children’s Hospital of Wenzhou Medical University, Wenzhou,, 325027 Zhejiang China; 5 Department of Neonatology, The Second Affiliated Hospital and Yuying Children’s Hospital, Wenzhou Medical University, Wenzhou,, 325027 China; 6 Chinese Orthopaedic Regenerative Medicine Society, Guangzhou, China; 7 The Second School of Medicine, Wenzhou Medical University, Wenzhou Medical University, Wenzhou, China

## Abstract

Oxidative stress-induced mitochondrial dysfunction is implicated in the pathogenesis of intervertebral disc degeneration (IVDD). Sirtuin 3 (SIRT3), a sirtuin family protein located in mitochondria, is essential for mitochondrial homeostasis; however, the role of SIRT3 in the process of IVDD has remained elusive. Here, we explored the expression of SIRT3 in IVDD in vivo and in vitro; we also explored the role of SIRT3 in senescence, apoptosis, and mitochondrial homeostasis under oxidative stress. We subsequently activated SIRT3 using honokiol to evaluate its therapeutic potential for IVDD. We assessed SIRT3 expression in degenerative nucleus pulposus (NP) tissues and oxidative stress-induced nucleus pulposus cells (NPCs). SIRT3 was knocked down by lentivirus and activated by honokiol to determine its role in oxidative stress-induced NPCs. The mechanism by which honokiol affected SIRT3 regulation was investigated in vitro, and the therapeutic potential of honokiol was assessed in vitro and in vivo. We found that the expression of SIRT3 decreased with IVDD, and SIRT3 knockdown reduced the tolerance of NPCs to oxidative stress. Honokiol (10 μM) improved the viability of NPCs under oxidative stress and promoted their properties of anti-oxidation, mitochondrial dynamics and mitophagy in a SIRT3-dependent manner. Furthermore, honokiol activated SIRT3 through the AMPK-PGC-1α signaling pathway. Moreover, honokiol treatment ameliorated IVDD in rats. Our study indicated that SIRT3 is involved in IVDD and showed the potential of the SIRT3 agonist honokiol for the treatment of IVDD.

## Disc degeneration: a potential defense against damaged discs

Molecules that counteract metabolic abnormalities in spinal tissue could help treat lower back pain associated with disc degeneration. In this condition, the cells that form the discs experience heightened oxidative stress, wherein mitochondria produce high levels of toxic compounds. Researchers in China led by Xiangyang Wang of the Second Affiliated Hospital of Wenzhou Medical University and Xiaolei Zhang of the Zhejiang Provincial Key Laboratory of Orthopedics have identified a molecular target that may enable doctors to prevent this damage. They determined that a protein called SIRT3 plays a critical role in mitigating oxidative stress, and that its loss renders stressed disc tissue especially vulnerable. The researchers also found that honokiol—a SIRT3-activating compound derived from magnolia tree bark—effectively prevents disc degeneration in rats. A similar treatment strategy could potentially prove beneficial in human patients.

## Introduction

Low-back pain pervasively affects up to 80% of adults at a certain period during their life time^1^. In general, intervertebral disc degeneration (IVDD) is considered to be the main cause of low-back pain^2^. During degeneration, nucleus pulposus cells (NPCs) exhibit dramatic molecular changes in extracellular matrix (ECM) metabolism, which depends on the quality of NPCs^3^.

Oxidative stress is common in degenerative IVDs resulting from age-related diseases, such as diabetes^4^. It has been reported that oxidative stress may induce disc cell apoptosis, senescence, and abnormal matrix metabolism^5^. Studies have demonstrated that mitochondrial dysfunction is involved in diverse degenerative diseases, including IVDD^6–8^, and dysfunctional mitochondria are the major source of reactive oxygen species (ROS) in cells. Thus, the maintenance of mitochondrial homeostasis is considered a therapeutic target for these diseases.

SIRT3 is a deacetylase that influences almost every major aspect of mitochondrial biology, including energy metabolism, ROS detoxification, mitochondrial dynamics, mitochondrial unfolded protein response, and mitophagy. SIRT3 deficiency may lead to mitochondrial dysfunction and increase the vulnerability of cells to oxidative stress^9,10^. Downregulation of SIRT3 has been implicated in various disorders including degenerative diseases^11–14^, whereas the role of SIRT3 in IVDD remains unknown.

Honokiol (HKL), a small molecular weight natural compound extracted from the bark of magnolia trees, has many pharmaceutical properties, such as analgesia, anti-inflammation, antitumor, and neuroprotection^15,16^. A recent study demonstrated that HKL is able to reverse myocardial hypertrophy by activating SIRT3^17^. Therefore, we hypothesize that HKL may block the development of IVDD via the upregulation of SIRT3.

In this study, we reported the expression of SIRT3 in IVDD and confirmed the role of SIRT3 in NPCs under oxidative stress; moreover, we activated SIRT3 using HKL to evaluate its therapeutic potential in IVDD. To our knowledge, this is the first report to describe the link between SIRT3 and IVDD and introduce HKL, a pharmacological agonist of SIRT3, for the treatment of IVDD.

## Materials and methods

### Ethics statement

All surgical interventions, treatments and postoperative animal care procedures were performed in strict accordance with the Animal Care and Use Committee of Wenzhou Medical University. Human nucleus pulposus tissue collection and experiments that involved human nucleus pulposus were approved by the Second Affiliated Hospital and Yuying Children’s Hospital of Wenzhou Medical University Ethics Committee and followed the guidelines of the Declaration of Helsinki^18^. The letter of ethical approval is provided in the Supplementary files.

### Reagents and antibodies

Honokiol was purchased from Meilunbio (Dalian, Liaoning, China), and its purity was ≥98%. The p16INK4α antibody, TBHP, and type II collagenases were obtained from Sigma-Aldrich (St. Louis, MO, USA). The primary antibodies of p-AMPK, PGC-1α, Mfn2, Drp1, Bnip3, and Bnip3L were acquired from Abcam (Cambridge, UK). The AMPK, SIRT3, and LC3 were supplied by CST (MA, USA). The Fis1 antibody was obtained from Genetex (Irvine, USA). The β-actin antibody, 4′, 6-diamidino-2-phenylindole (DAPI), malondialdehyde (MDA) assay kit, and total superoxide dismutase assay kit with NBT were purchased from Beyotime (Shanghai, China). Alexa-Fluor-488- and Alexa-Fluor-594-tagged second antibodies were obtained from Abcam. The catalase assay kit was obtained from Nanjing Jiancheng Bioengineering Institute (Nanjing, China). Compound C was obtained from Selleck (Houston, USA). The Bnip3L antibody, PGC-1α siRNA (sc-72151), siRNA transfection medium (sc-36868), and siRNA transfection Reagent (sc-29528) were purchased from Santa Cruz Biotechnology (Dallas, TX, USA). The reagents for cell culture were purchased from Gibco (Grand Island, NY, USA).

### Human nucleus pulposus collection

To investigate the relationship between SIRT3 and the degree of disc degeneration, 16 NP tissues from IVDD patients (2 males and 2 females, age range from 46 to 53 years, grade II; 3 males and 3 females, age range from 46 to 54 years, grade III; 2 males and 3 females, age range from 46 to 56 years, grade V) were collected to perform further experiments according to the Pfirrmann grading scale^19^. In our study, there were no other complications related to IVDD, such as diabetes mellitus, in the patients whose NP tissues were collected during surgery. The collected nucleus pulposus tissues were lysed and subsequently used for Western blotting.

### Rat nucleus pulposus cell isolation and culture

Gel-like NP tissues were collected from the tails of 2-week-old pups (Sprague–Dawley rats, either sex). The NP tissues were digested in 0.2% type II collagenase (Sigma) for 4 h at 37 °C. After washing with PBS, the digested tissues were transferred to DMEM/F12 (Gibco, Invitrogen, Grand Island, NY) with 15% fetal bovine serum (FBS; Gibco, Invitrogen, Grand Island, NY) and antibiotics (1% streptomycin/penicillin) in an incubator at 5% CO_2_ and 37 °C. When confluent, the cells were passaged after trypsinizing with 0.25% Trypsin-EDTA (Gibco, Invitrogen) and were replanted in 10-cm culture plates at the appropriate density. We used the first three passages of cells in our experiments.

### Treatment protocol of cell culture

To investigate the SIRT3 expression level in NPCs during oxidative stress, NPCs were treated with different concentrations of TBHP (0, 25, 50, or 75 μM) for 3 h and with the same concentration of TBHP (75 μM) for different times (0, 1, 2, or 3 h). To determine the effect of HKL on the viability of NPCs, cells were incubated with increasing concentrations (0.1–20 μM) of HKL for 24 h. Cells were pretreated with different concentrations of HKL (0.1–10 μM) for 2 h prior to the additional administration of TBHP to explore the effects of HKL in NPCs. To investigate the role of HKL in mitochondrial protection, NPCs were pretreated with 2.5 μM of Compound C (Selleck, Houston, USA) for 4 h prior to the administration of HKL and TBHP.

### Cell viability assay

According to the manufacturer’s protocol, cell viability was detected using the cell counting kit-8 (CCK-8; Dojindo Co., Kumamoto, Japan). NPCs were treated with HKL, TBHP, and lentivirus as described in Fig. 3. After washing the cells with PBS, 100 μl of DMEM/F12 that contained 10 μl of CCK-8 solution was added to each well. The plate was subsequently incubated for approximately 1 h. The absorbance of the wells was measured using a microplate reader at 450 nm.

### siRNA and lentivirus transfection

siRNA for PGC-1α was obtained from Santa Cruz Biotechnology (sc-72151; Dallas, TX, USA). The NPCs were transfected with PGC-1α siRNA according to the manufacturer’s protocol.

When 30–50% confluence was reached, the NPCs were transfected with lentivirus (GeneChem, Shanghai, China) at a multiplicity of infection (MOI) of 100. The culture medium was changed after 12 h of transfection, when more than 95% of the cells were alive. After 3 days, all transfected cells were passaged for further experiments. The expression of PGC-1α and SIRT3 was quantified by Western blot analysis.

### Transmission electron microscopy

After being fixed in 2.5% glutaraldehyde overnight, NPCs were fixed in 2% osmium tetroxide for 1 h and stained with 2% uranyl acetate for 1 h. Prior to being embedded in araldite and cut into semithin sections, the samples were dehydrated in an ascending series of acetone. Then, semithin sections were stained with toluidine blue to locate cells prior to observation with a transmission electron microscope (Hitachi, Tokyo, Japan).

### Western blot assay

NPCs were lysed in ice-cold RIPA with 1 mM PMSF (phenylmethanesulfonyl fluoride, Beyotime). The protein concentrations of the samples were measured using the BCA protein assay kit (Beyotime). The proteins of NPCs were separated via sodium dodecyl sulfate–polyacrylamide gel electrophoresis (SDS–PAGE) and were transferred to a polyvinylidene difluoride membrane (Millipore, USA) followed by blocking with 5% nonfat milk. The bands were subsequently probed with primary antibodies specific to SIRT3 (1:1000), p-AMPK (1:500), AMPK (1:1000), PGC-1α (1:1000), Mfn2 (1:1000), Bnip3 (1:1000), Bnip3 L (1:1000), MMP-13 (1:1000), LC3 (1:1000), Drp1 (1:1000), Fis1 (1:1000), and β-actin (1:1000) overnight at 4 °C, prior to incubation with the respective secondary antibodies. Finally, the intensity of the bands was quantified using Image Lab 3.0 software (Bio-Rad).

### Immunofluorescence

Samples were blocked with 10% goat serum for 30 min at room temperature. Primary antibodies against SIRT3 (1:200), LC3 (1:100), Bnip3L (1:100), collagen II and MMP-3 were applied to the incubation of the samples at 4 °C overnight. The slides were subsequently incubated with fluorescein isothiocyanate- or tetramethyl rhodamine isothiocyanate-conjugated secondary antibodies for 1 h and labeled with DAPI for 5 min. The slides were then observed using a fluorescence microscope (Olympus Inc., Tokyo, Japan).

### Nucleus morphology assay

The nucleus morphology was dyed using Hoechst 33342 (Yeasen, Shanghai, China) according to the manufacturer’s instructions and was observed with a fluorescence microscope (Olympus Inc., Tokyo, Japan).

### MitoSOX assay

To evaluate mitochondrial ROS, an immunofluorescence probe was performed. Briefly, the rat NPCs were seeded on a cover-glass bottom dish and treated as described. The cells were subsequently incubated with 5 μM MitoSOX for 15 min at 37 °C. The NPCs were then washed twice with PBS and incubated with Hoechst 33342 solution at 37 °C for 15 min. Images were acquired using a fluorescence microscope with an exposure time of 500 ms and an iso of 200 (Olympus Inc., Tokyo, Japan).

### Mitochondrial membrane potential assay

The mitochondrial transmembrane potential (MMP) was detected using a red-fluorescent dye, MitoTracker red CMXRos (Molecular Probes^TM^, Thermo Fisher Scientific Inc.), which stains mitochondria in live cells and accumulates in an MMP-dependent manner, at a concentration of 50 nM for 30 min at 37 °C. The nuclei were subsequently stained with Hoechst 33342 dye for 10 min at 37 °C. The samples were observed with a fluorescence microscope (Olympus Inc., Tokyo, Japan), and the fluorescence intensity was quantified using ImageJ software 2.1 (Bethesda, MD, USA).

### Sa-β-gal staining

After being washed twice with PBS, cells on plates were fixed with 0.2% glutaraldehyde for 10 min at room temperature. The cells were subsequently stained with X-gal staining solution overnight at pH 6.0. Images were captured using an Olympus IX71 microscope, and the percentages of SA-β-gal-positive cells were quantified for statistical analysis.

### Analysis of MDA and enzymatic activity of SOD

The MDA level and SOD enzymatic activity in the NPCs were analyzed using an MDA assay kit and SOD assay kit (Beyotime Institute of Biotechnology, Shanghai, China) according to the manufacturer’s instructions.

### Surgical procedure

SD Rats (200–250 g, male, *n* = 24) were randomly divided into three groups (*n* = 8 per group, including the vehicle group, IVDD group and HKL group), as previously described. The HKL group underwent a model operation similar to that performed in the IVDD group. After anesthetization with 2% (w/v) pentobarbital (40 mg/kg), the specific level of the rat tail disc (Co7/8) was located by palpation on the coccygeal vertebrae, and the disc location was confirmed with an X-ray radiograph. Needles (27 G) were applied to perpendicularly puncture the AF through the tail skin, and the depth of the puncture was 4 mm, according to methods described in a previous study^20^. The needles were maintained in the disc for 1 min.

Prior to the surgery, the rats were randomly divided into three groups: SHAM + vehicle, IVDD + vehicle, and IVDD + HKL. The rats in the HKL group were orally administered HKL at a concentration of 40 mg/kg (4 mg mL^−1^ of HKL was suspended in 0.5% CMC-Na solution) for 1 week, while the other rats were administered a 0.5% CMC-Na solution. After the surgery, the rats were treated as described until sacrifice.

### MRI methods

The IVD signal and structural changes were assessed with MRI on sagittal T2-weighted images using a 3.0-T clinical magnet (Philips Intera Achieva 3.0MR). The parameters of T2-weighted imaging were based upon methods described in a previous study^21^. According to the MRIs, the degree of IVDD in the rats was evaluated with the Pfirrmann grading system^19^.

### Histopathologic analysis

Rats were executed with an intraperitoneal lethal dose injection of chloral hydrate, and the tails were harvested. After being fixed in formaldehyde and decalcified, the specimens were dehydrated and embedded in paraffin. The tissues were cut into 5 μM sections. Following the HE staining, the morphology of the IVD was evaluated with a grading system as previously described^22^. The SIRT3 expression level in the degenerated discs was detected by immunofluorescence, and the fluorescence intensity was quantified using ImageJ software 2.1.

### Statistical analysis

All experiments were performed at least three times. The results are expressed as the mean ± S.D. Raw statistical analyses were processed using SPSS statistical software program 20.0. Data were analyzed by one-way analysis of variance (ANOVA) followed by the Tukey’s test for comparisons between the control and treatment groups. Nonparametric data (Pfirrmann MRI grade scores) were analyzed by the Kruskal–Wallis *H* test. Differences were considered statistically significant when *P* < 0.05.

## Results

### SIRT3 expression was decreased in human degenerated NP tissues and increased in TBHP-induced NPCs

**Fig. 1:**
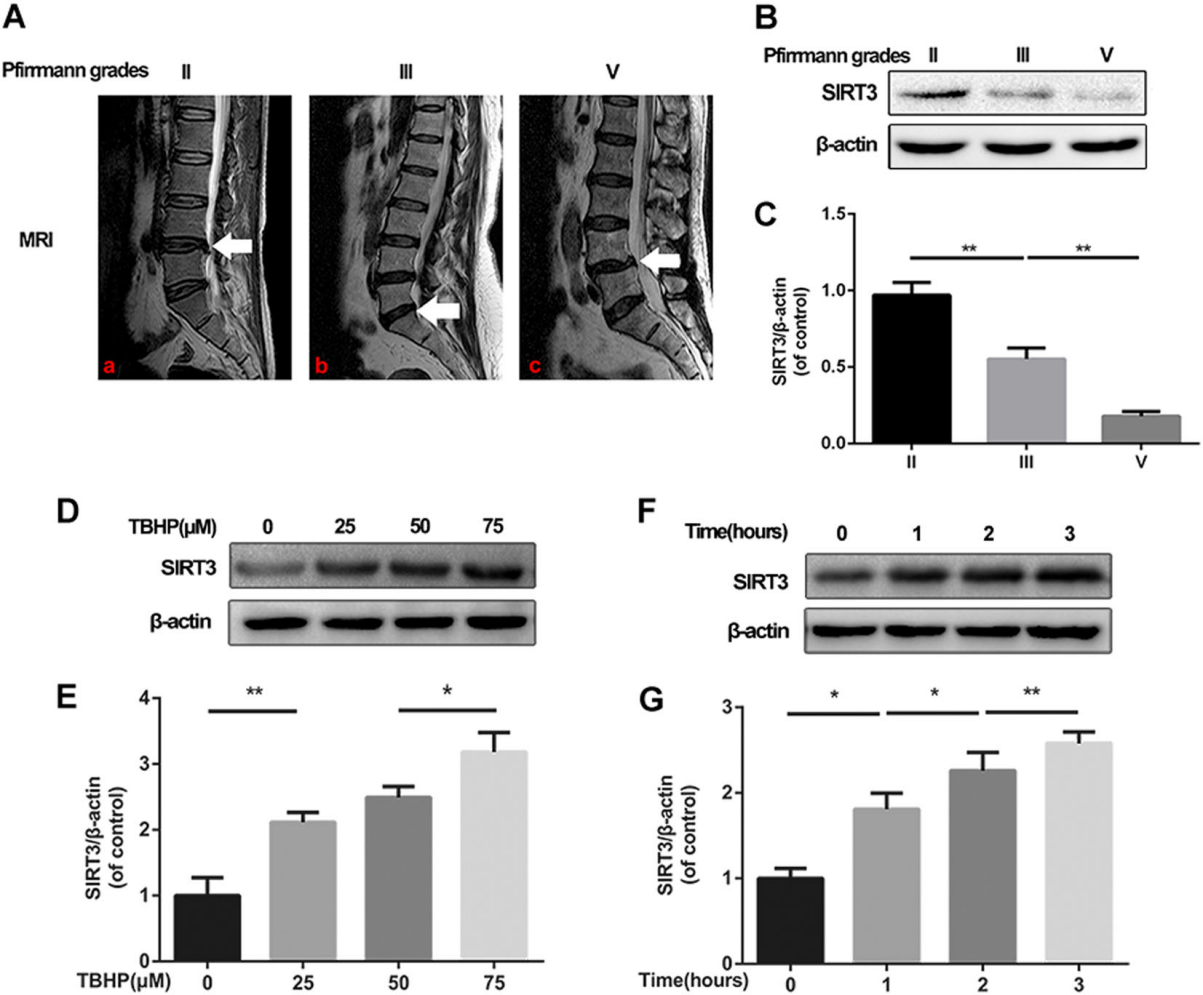
The expression of SIRT3 decreases in degenerated human disc tissues and increases in TBHP-treated rat NPCs. **a, b** Representative MR images of three different degrees of IVDD patients. **a** Pfirrmann grade II patient, male, 46-years old, lumbar disc herniation. **b** Pfirrmann grade III patient, female, 53-years old, lumbar disc herniation. **c** Pfirrmann grade V patient, female, 51-years old, lumbar disc herniation. Nucleus pulposus samples from different patients of ages were collected for the measurement of SIRT3, with consistency in the degree of intervertebral disc degeneration. Western blot analysis and bar diagrams of SIRT3 showed that the protein levels of SIRT3 decreased in an age-dependent manner. **c** Representative Western blots and bar diagram of SIRT3 levels in each human NP tissue group (Pfirrmann grades II, III, and V). **d, e** The effect of different concentrations of TBHP on rat NPCs. **f, g** The effect of different durations of treatment with TBHP on rat NPCs. All experiments were performed in duplicates, and data are reported as the mean ± SD. ^*^P < 0.05, ^**^P < 0.01

Previous studies have shown that SIRT3 expression is significantly decreased in degenerative diseases. To determine whether SIRT3 expression was correlated with IVDD, human NP tissues of patients from different degenerative degrees were harvested to measure the SIRT3 level by Western blotting. We collected 5 grade II, 6 grade III, and 5 grade V NP samples classified according to the Pfirrmann grade system^19^ (Fig. 1a). As shown in Fig. 1b, c, we found that the expression level of SIRT3 in NP samples decreased with the degree of disc degeneration. However, oxidative stress induced by TBHP upregulated the protein level of SIRT3 in a dose- and time-dependent manner (Fig. 1d–g). Collagen II represents the ability of ECM synthesis, and MMP-3 is a marker of ECM degradation. As shown in Supplementary Figure 1, we found that oxidative stress caused a decreased level of collagen II and increased MMP-3 expression in NPCs, indicating that oxidative stress induced by TBHP aggravated NPC degeneration. Taken together, our data showed that SIRT3 might be associated with NPC degeneration.

### SIRT3 regulates senescence and apoptosis in TBHP-treated NPCs under oxidative stress

**Fig. 2:**
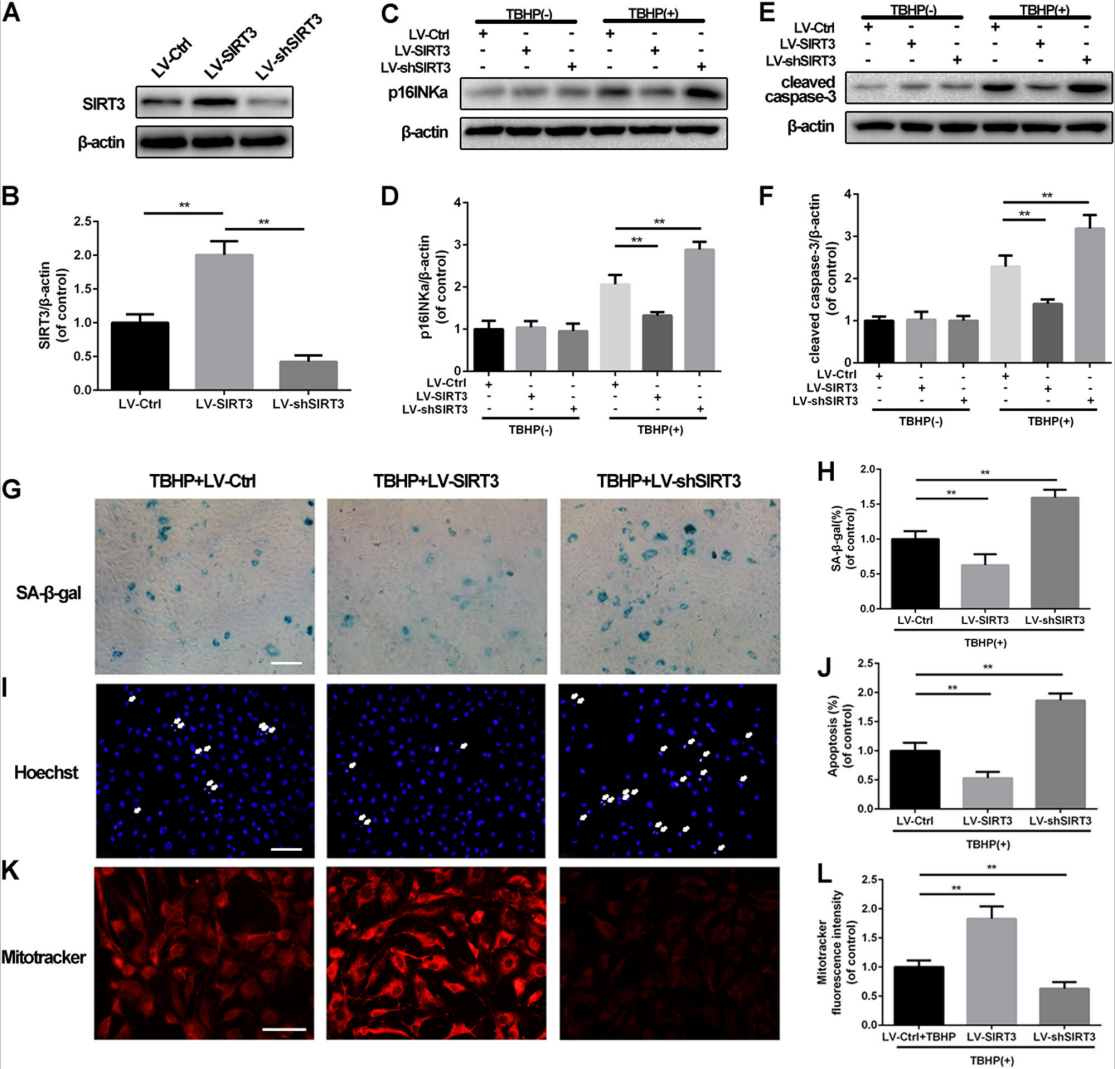
The effects of SIRT3 knockdown and overexpression on apoptosis, senescence, and mitochondrial dysfunction under oxidative stress in rat NPCs. **a**, **b** Successful knockdown of SIRT3 in rat NPCs was established using LV-shSIRT3. **c**, **d** The effects of SIRT3 on the cleaved caspase-3 levels under conditions of oxidative stress. **e**, **f** The effects of SIRT3 on the p16INKa levels under conditions of oxidative stress. **g**, **h** Representative images of SA-β-gal staining of NPCs from different groups. Scale bar: 50 μM. **i**, **j** The nucleus morphology was detected using Hoechst 33342. Scale bar: 50 μM. **k**, **l** Mitochondrial membrane potential of rat NPCs was measured using MitoTracker red CMXRos. Scale bar: 50 μM. All experiments were performed in duplicate, and data are reported as the mean ± SD. ^*^P < 0.05, ^**^P < 0.01

Because the expression of SIRT3 in degenerative human NPCs was not consistent with that in rat NPCs treated with TBHP, we knocked down and overexpressed SIRT3 to determine whether SIRT3 acted as a protective mediator against oxidative stress in NPCs. First, we established SIRT3 knockdown and SIRT3 overexpression cell models (Fig. 2a, b). We subsequently detected the role of SIRT3 in TBHP-induced senescence and apoptosis in NPCs. The results of the Western blot analysis showed that SIRT3 knockdown augmented the senescence marker protein expression of p16INKa, while SIRT3 overexpression suppressed the p16INKa level under TBHP-induced oxidative stress conditions (Fig. 2c, d). As shown in Fig. 2e, f, under oxidative stress induced by TBHP, SIRT3 knockdown enhanced cleaved caspase-3 expression, while SIRT3 overexpression downregulated the SIRT3 level in NPCs. SA-β-gal staining and nucleus staining are common methods used to detect senescence and apoptosis, respectively. Compared to the NPCs transfected with scramble lentivirus, the percentage of SA-β-gal-positive staining cells in the SIRT3 knockdown group was increased, whereas SIRT3 overexpression suppressed the percentage of senescent cells under conditions of oxidative stress (Fig. 2g, h). Pathological changes in nucleus morphology, such as karyopyknosis, karyorrhexis and necrosis, are characteristic of apoptosis. As shown in Fig. 2i, j, the percentage of apoptotic cells was reduced in the SIRT3 overexpression group and increased in the SIRT3 knockdown group. Collectively, our data demonstrated that SIRT3 protects NPCs from oxidative stress-induced senescence and apoptosis.

### SIRT3 promotes mitochondrial homeostasis in TBHP-treated NPCs

As mitochondria are responsible for both senescence and apoptosis, SIRT3 is also closely related to mitochondrial quality; thus, we evaluated whether SIRT3 could affect mitochondrial quality in NPCs. MitoTracker red CMXRos, which accumulates in the mitochondrial membrane in a potential-dependent manner, was used to detect the early stage of mitochondrial dysfunction. As shown in Fig. 2k, l, SIRT3 knockdown reduced the degree of MitoTracker fluorescence, while SIRT3 overexpression promoted MitoTracker fluorescence intensity in NPCs under conditions of oxidative stress, indicating that SIRT3 exerts a protective effect on mitochondrial homeostasis against oxidative stress.

### Activation of SIRT3 by honokiol suppresses senescence and apoptosis in TBHP-treated NPCs

**Fig. 3:**
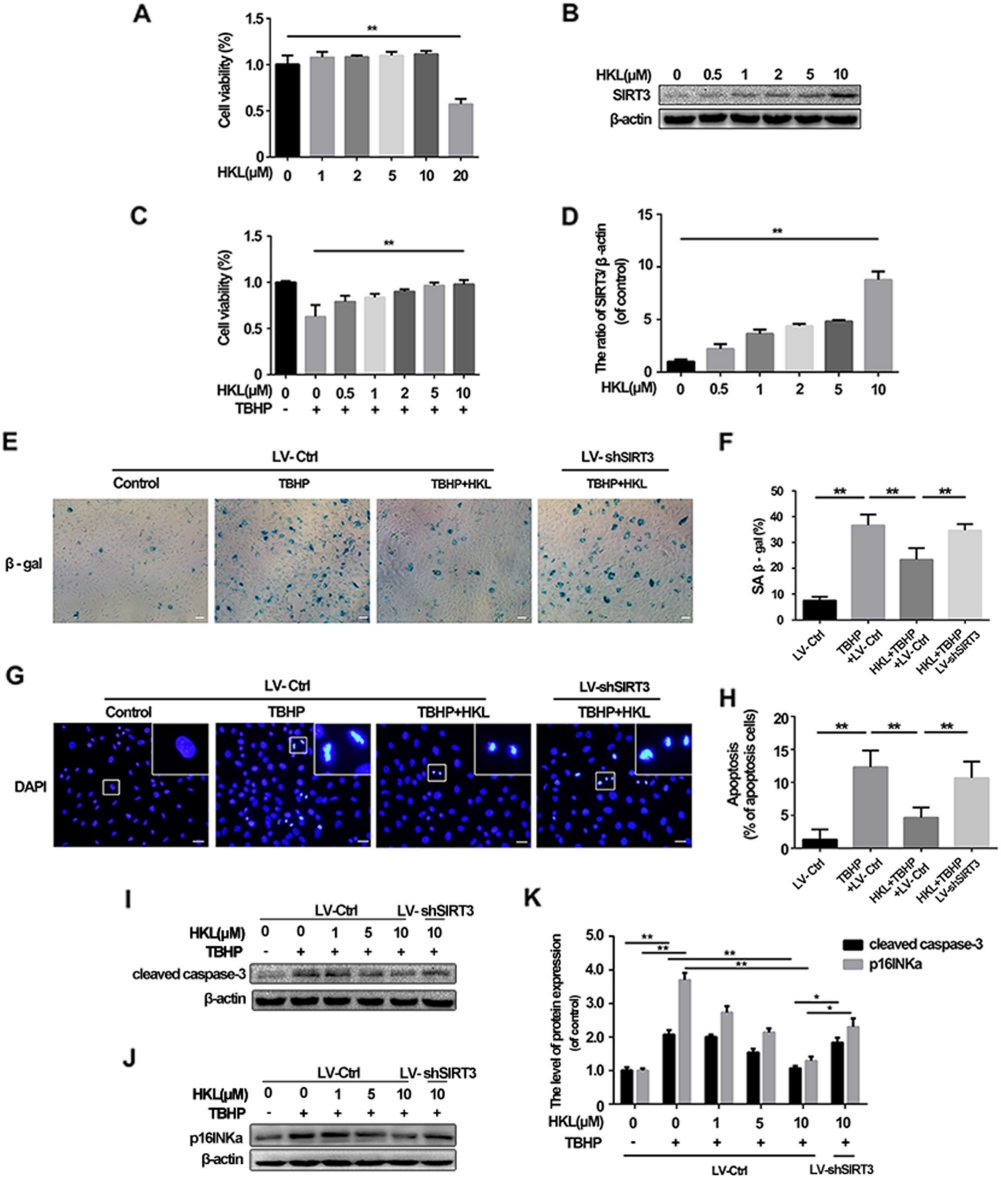
HKL suppresses apoptosis and senescence induced by TBHP by activating SIRT3 in rat NPCs. **a** NPCs were treated with different doses of HKL for 24 h. **b, c** NPCs were treated with different doses of HKL as indicated. Cell lysate was harvested and analyzed for the expression of SIRT3 using Western blots. **d** NPCs, pretreated with different doses of HKL, were administered with TBHP. **e, f** SIRT3 knockdown inhibited the capacity of the anti-senescence of HKL in NPCs. Scale bar 100 μM. **g, h** Fluorescence microscopy of Hoechst 33342 staining for morphological assessment of apoptosis. Apoptotic nuclei were characterized by condensed chromatin and nucleus fragmentation. Scale bar, 50 μM. **i–k** The expressions of cleaved caspase-3 and p16INKa were detected by western blotting. All experiments were performed in duplicate, and data are reported as the mean ± SD. ^*^P < 0.05, ^**^P < 0.01

As SIRT3 knockdown exacerbated oxidative stress-induced damage in NPCs, we hypothesized that SIRT3 activation could exert protective effects in oxidative stress-induced NPCs. Therefore, we used HKL, a well demonstrated SIRT3 agonist, to activate SIRT3 in NPCs. First, we determined the safe (nontoxic) concentration of HKL in NPCs. NPCs were administered HKL for 24 h at different concentrations (from 0 to 20 μM). As shown in Fig. 3a, HKL was not cytotoxic to the NPCs when the concentration was less than (or equal to) 10 μM. Moreover, HKL was able to activate SIRT3 in NPCs and rescue cell viability under conditions of oxidative stress in a dose-dependent manner (Fig. 3b–d).

We subsequently evaluated the effects of HKL on senescence and apoptosis via SIRT3 activation. We found that the percentage of SA-β-gal-positive cells was increased in the oxidative stress group when these cells transfected with scramble lentivirus, while HKL treatment could reduce the proportion of senescent NPCs via SIRT3 activation (Fig. 3e, f), which was further confirmed by the Western blot analysis of p16INKa (Fig. 3j, k). Apoptosis detection coincided with the results of senescence, as shown by Hoechst 33342 staining and cleaved caspase-3 Western blotting (Fig. 3g–i, k). Together, these results demonstrate that HKL could upregulate SIRT3 in a dose-dependent manner and that SIRT3 activation by HKL suppresses oxidative stress-induced senescence and apoptosis in NPCs.

### HKL preserves mitochondrial anti-oxidation capacity via SIRT3 activation

**Fig. 4:**
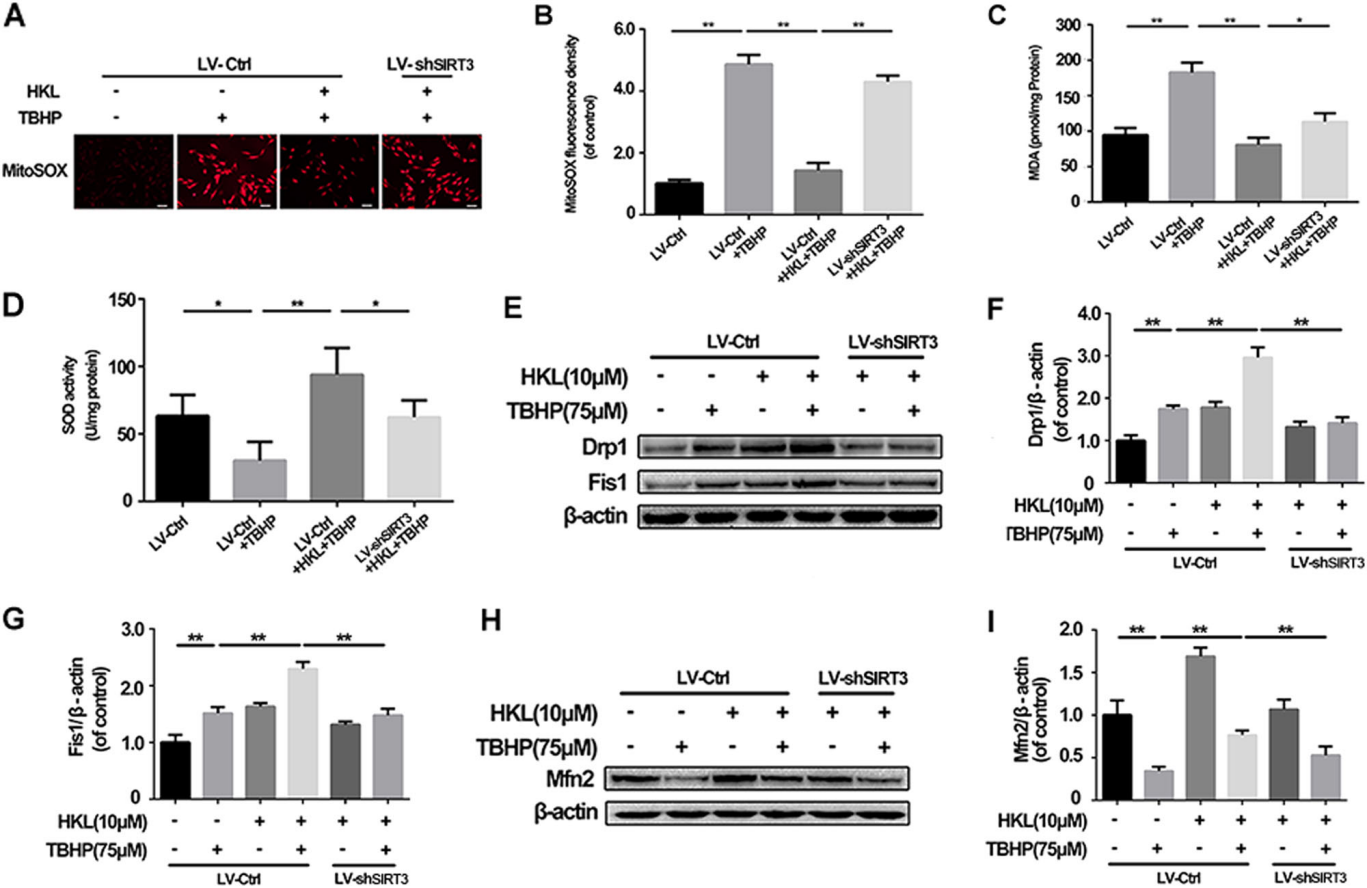
HKL promotes the capacity of mitochondrial anti-oxidation and dynamics in a SIRT3-dependent manner in rat NPCs. **a, b** Representative micrographs showed MitoSOX red fluorescence in no knockdown or SIRT3 knockdown in NPCs with or without treatment of HKL or TBHP. Scale bar: 20 μM. **c** Regulation of MDA in NPCs under conditions of oxidative stress with or without HKL and SIRT3 knockdown. **d** TBHP reduced activity of SOD in NPCs, which was increased with the treatment of HKL by activating SIRT3. **e–g** Effect of HKL on expression of the mitochondrial fission dynamic markers Drp1 and Fis1. **h, i** Diagrams show the effect of HKL on the expression of the mitochondrial dynamic marker Mfn2. All experiments were performed in duplicate, and data are reported as the mean ± SD. ^*^P < 0.05, ^**^P < 0.01

To determine how HKL exerts the capacity of mitochondrial protection in NPCs, the content of O_2_·^−^ was measured via a MitoSOX assay. As shown in Fig. 4a, b, the MitoSOX fluorescence intensity was increased in the TBHP group, while HKL treatment reduced the MitoSOX fluorescence intensity in the NPCs under oxidative stress, and SIRT3 knockdown diminished the regulative effect of HKL on superoxide anion (O_2_·^−^). MDA, a cell membrane lipid peroxidation product, is commonly used as an indicator of intracellular oxidative stress. In the current study, TBHP markedly increased the content of MDA, which was reversed by HKL in a SIRT3-dependent manner (Fig. 4c). Superoxide dismutase (SOD) is able to convert O_2_·^−^ to hydrogen peroxide, which is subsequently detoxified into H_2_O and O_2_. As shown in Fig. 4d, HKL significantly rescued the enzymatic activity of SOD in the TBHP-treated NPCs. These effects of HKL on the capacity of anti-oxidation could be abolished by SIRT3 knockdown. Together, our results show that HKL can preserve mitochondrial anti-oxidation in TBHP-treated NPCs, and its effect may be related to SIRT3 activation.

### HKL promotes mitochondrial dynamics via SIRT3 activation

Under stressful conditions, timely mitochondrial fission and fusion are essential to maintain the mitochondrial quality^23^. We assessed the expression of Drp1 and Fis1, which are essential mediators in mitochondrial fission, and the expression of Mfn2, which is indispensable for mitochondrial fusion. As shown in Fig. 4e–g, increased levels of Drp1 and Fis1 were identified in the NPCs under conditions of TBHP-induced oxidative stress. In addition, the levels of Drp1 and Fis1 could be further elevated by pretreatment with HKL. TBHP could reduce the level of Mfn2, whereas HKL could alleviate the reduction in Mfn2 expression (Fig. 4h, i). These effects of HKL on mitochondrial dynamics were suppressed by SIRT3 knockdown. Thus, these findings suggest that HKL may promote mitochondrial dynamics via SIRT3 activation.

### HKL activates SIRT3 to induce mitophagy in response to oxidative stress

**Fig. 5:**
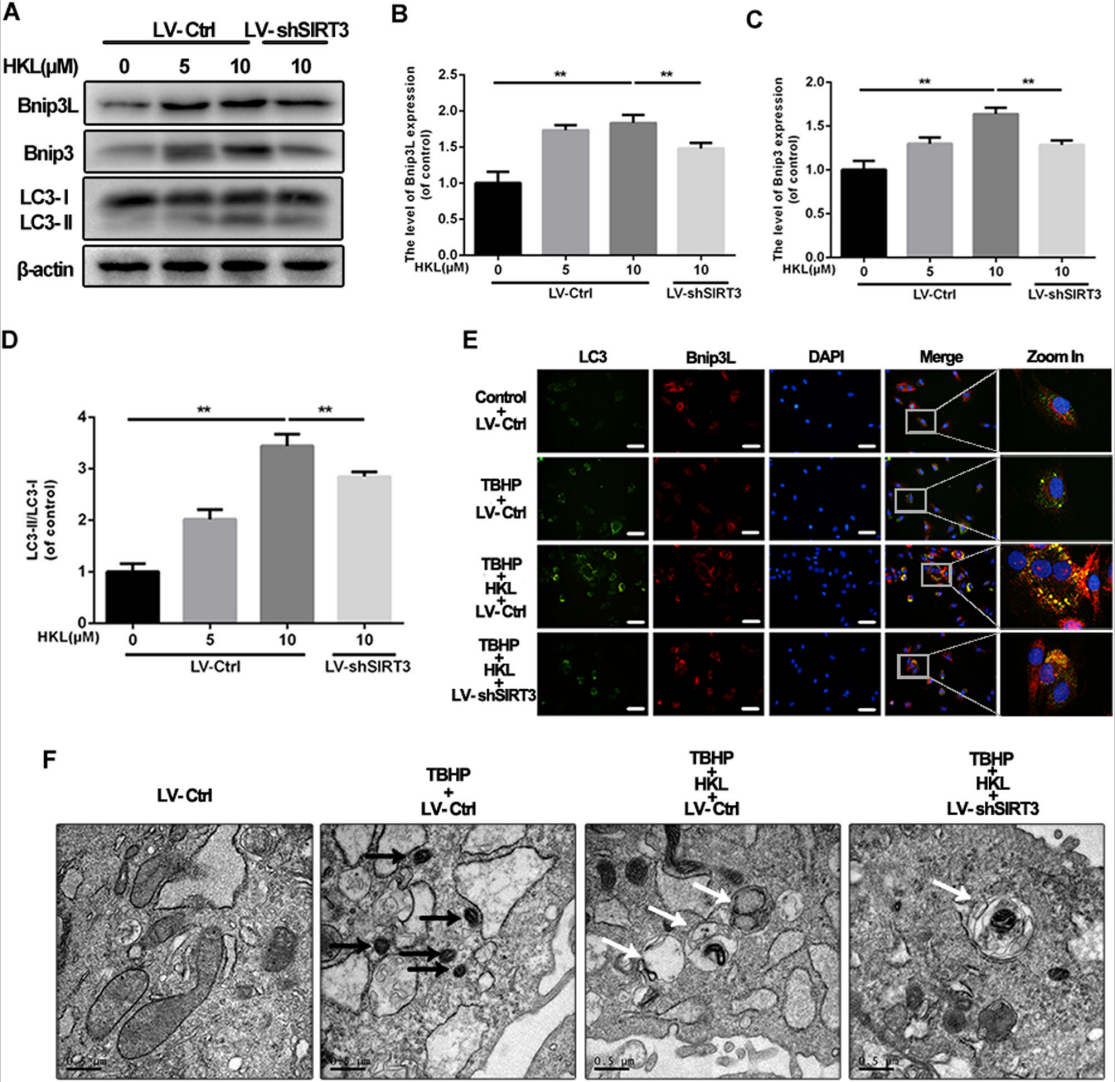
HKL promotes mitophagy in an SIRT3-dependent manner in rat NPCs. **a–d** western blots of mitophagy markers, including Bnip3L, Bnip3, and LC3. HKL increased the mitophagy markers Nix and Bnip3 and the ratio of LC3-II/LC3-I by activating SIRT3. **e** The representative colocalization of LC3 and Bnip3L is detected by immunofluorescence staining. Scale bar: 50 μM. **f** Transmission electron microscopy was used to detect the mitophagosomes (×25,000) in nucleus pulposus cells. (Black arrow: mitochondria; white arrow: mitophagy). All experiments were performed in duplicate, and data are reported as the mean ± SD. ^*^P < 0.05, ^**^P < 0.01

Mitophagy is essential for quality control and impairment in the recovery of mitochondria; thus, the following experiments were performed to determine whether HKL may regulate mitophagy in NPCs under conditions of oxidative stress. As shown in Fig. 5a–d, the expressions of core mitophagic indicators, such as Bnip3 and Bnip3L, and the ratio of LC3-II/LC3-I were increased in the NPCs treated with increasing concentrations of HKL. However, SIRT3 knockdown compromised the effects of HKL on mitophagy, indicating HKL promoted mitophagy by activating SIRT3. These findings were further proved by immunofluorescence analysis to detect the colocalization of LC3 and Bnip3L, which showed more double-positive puncta with treatment with HKL under conditions of oxidative stress (Fig. 5e). Furthermore, the results of transmission electron microscopy showed that HKL generated more mitophagic vesicles that contained impaired mitochondria under oxidative stress, which could be inhibited by SIRT3 knockdown (Fig. 5f). These data suggest that HKL can facilitate mitophagy in TBHP-treated NPCs by enhancing SIRT3.

### AMPK-PGC-1α-SIRT3 signaling pathway is activated by HKL in NPCs

**Fig. 6:**
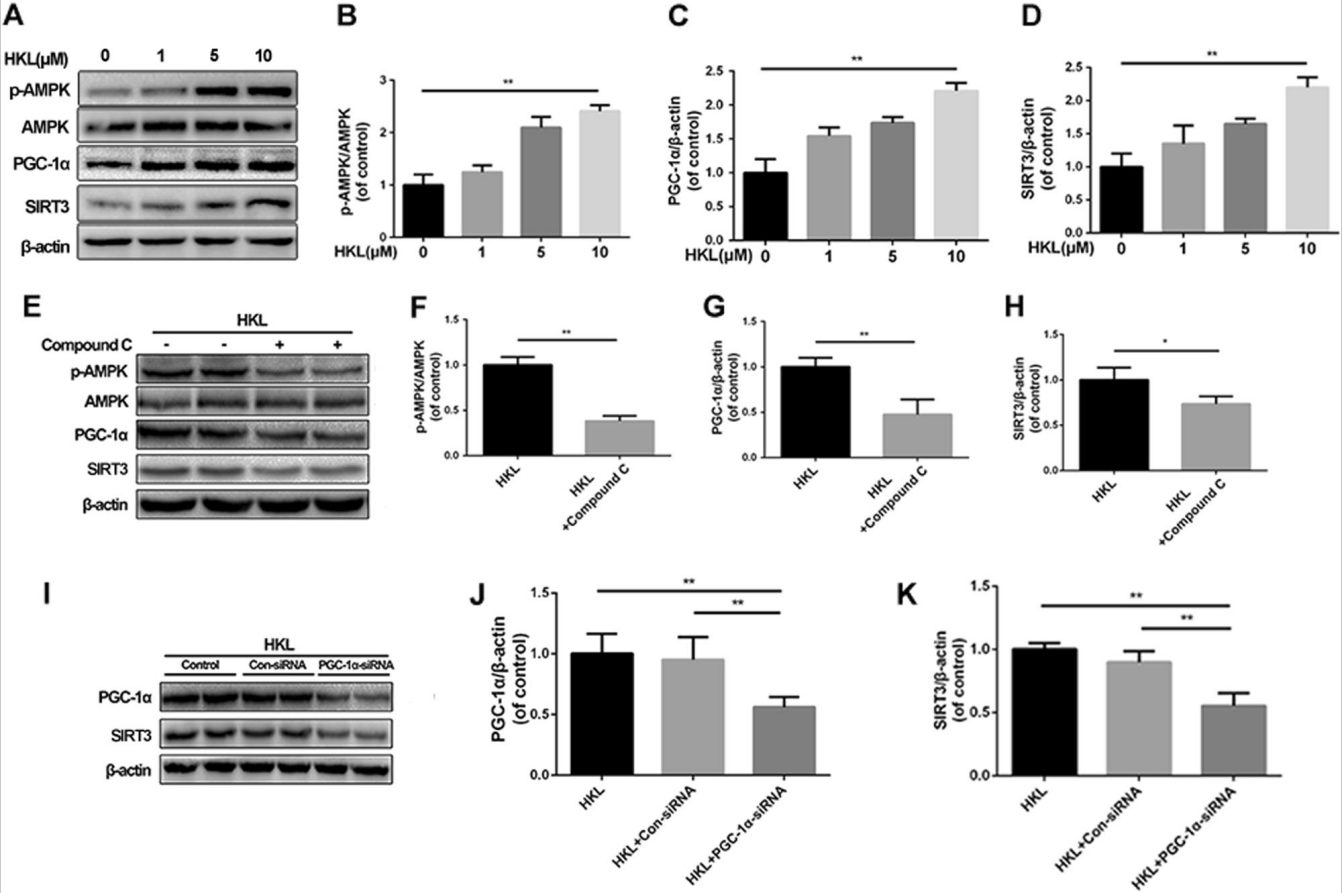
HKL stimulates AMPK-PGC-1α-SIRT3 signaling pathway in rat NPCs. **a–d** Following HKL administration, the expressions of p-AMPK, AMPK, PGC-1α, and SIRT3 were measured by Western blotting. **e–h** NPCs were pretreated with HKL (10 μM) for 24 h and then treated with or without Compound C, the selected AMPK inhibitor at 2.5 μM for an additional 4 h. Cell lysate was harvested to detect the expression levels of p-AMPK, AMPK, PGC-1α, and SIRT3 by Western blotting. **i–k** After 24 h post-transfection with control siRNA and PGC-1α siRNA, cells were pretreated with HKL at 10 μM for 24 h. All experiments were performed in duplicate, and data are reported as the mean ± SD. ^*^P < 0.05, ^**^P < 0.01

To explore how HKL regulates SIRT3 expression in NPCs, we detected the pathway related to SIRT3 regulation. Our results showed that HKL could promote the ratio of p-AMPK to AMPK and the levels of PGC-1α and SIRT3 in a dose-dependent manner in NPCs (Fig. 6a–d). NPCs were subsequently administered Compound C to inhibit the phosphorylation of AMPK prior to HKL administration. Compared with the control group, Compound C reduced the ratio of p-AMPK to AMPK with decreasing levels of PGC-1α and SIRT3, indicating that PGC-1α and SIRT3 were regulated by AMPK (Fig. 6e–h). We subsequently silenced PGC-1α to determine whether it was able to regulate SIRT3 in HKL-treated NPCs. As shown in Fig. 6i–k, with the administration of HKL, NPCs displayed decreased expression of SIRT3 in the PGC-1α-siRNA group, suggesting that SIRT3 is regulated by PGC-1α in HKL-treated NPCs. These data suggest that HKL can effectively upregulate SIRT3 expression via the AMPK-PGC-1α-SIRT3 signaling pathway in NPCs.

### HKL ameliorates puncture-induced intervertebral disc degeneration in rats

**Fig. 7:**
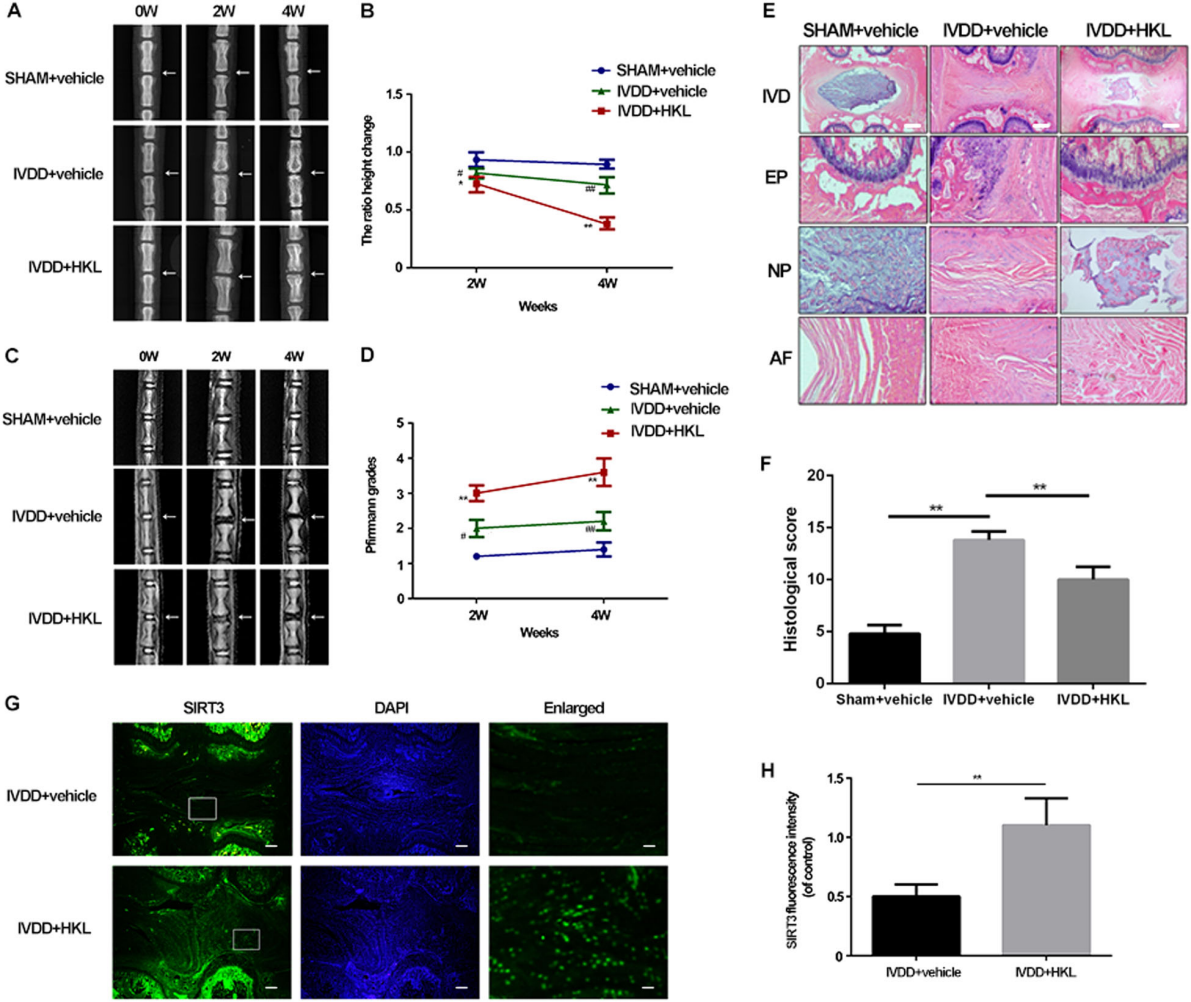
HKL alleviates puncture-induced disc degeneration of rats in vivo. **a** Radiographic images of rat tails with a needle-punctured disc at 2 and 4 weeks after surgery (white arrows). **b** Disc height index (DHI) of rat tail. **c, d** T2-weighted MRI of a rat tail with a needle-punctured disc preoperatively and at 2 and 4 weeks postoperatively. The Pfirrmann MRI grade scores were acquired from three groups at weeks 2 and 4. **e, f** The morphology of the disc was examined using H&E staining. Scale bar: 100 μM. **g, h** Immunofluorescence images of SIRT3 expression in the rat disc samples. Scale bar 100 μM. Each group consisted of 12 rats. ^*^P < 0.05, ^**^P < 0.01 compared with the control group. ^#^*P* < 0.05, ^##^*P* < 0.01 compared with the IVDD + vehicle group

On the basis of the results obtained in vitro, we subsequently assessed whether HKL could be used for intervertebral disc degeneration therapy in vivo. Caudal discs of rats were punctured to induce intervertebral disc degeneration, and radiographic images and MRIs were obtained at 0, 2, and 4 weeks after the disc puncture procedure and HKL treatment. As shown in Fig. 7a–d, prior to the puncture, there was no sign of disc degeneration on the X-rays or the MRIs. After two weeks, the IVDD group showed a loss of disc height and disorder of the structure of punctured discs. After 4 weeks, the differences between the control group and the IVDD group became more obvious. However, the administration of HKL partly alleviated the collapsed disc space and the loss of the MRI signal.

HE staining was also applied to evaluate the morphological changes of the intervertebral disc after HKL treatment. As shown in Fig. 7e, f, compared with the SHAM group, disorganized and hypocellular fibrocartilaginous tissues were found to replace the NP in the IVDD + vehicle group. Furthermore, the border between the AF and the NP was interrupted, which indicates that puncture resulted in severe disc degeneration. The layered structure of the AF was more distinct and the NP was larger in the IVDD + HKL group than in the IVDD + vehicle group, and the boundary between the AF and the NP was also preserved in the IVDD + HKL group, indicating HKL could ameliorate the disc structure destruction in puncture-induced IVDD in rats. In addition, a disorganized endplate (EP) was identified in the IVDD + vehicle group, while the morphology of the EP was relatively regular in the IVDD + HKL group. Moreover, SIRT3 immunohistofluorescence showed that HKL could increase the expression of SIRT3 in the rat NP, which suggests that HKL could be used as a SIRT3 agonist in vivo (Fig. 7g, h).

Together, these findings suggest that HKL treatment upregulates the level of SIRT3 in vivo and ameliorates puncture-induced intervertebral disc degeneration in rats.

## Discussion

**Fig. 8:**
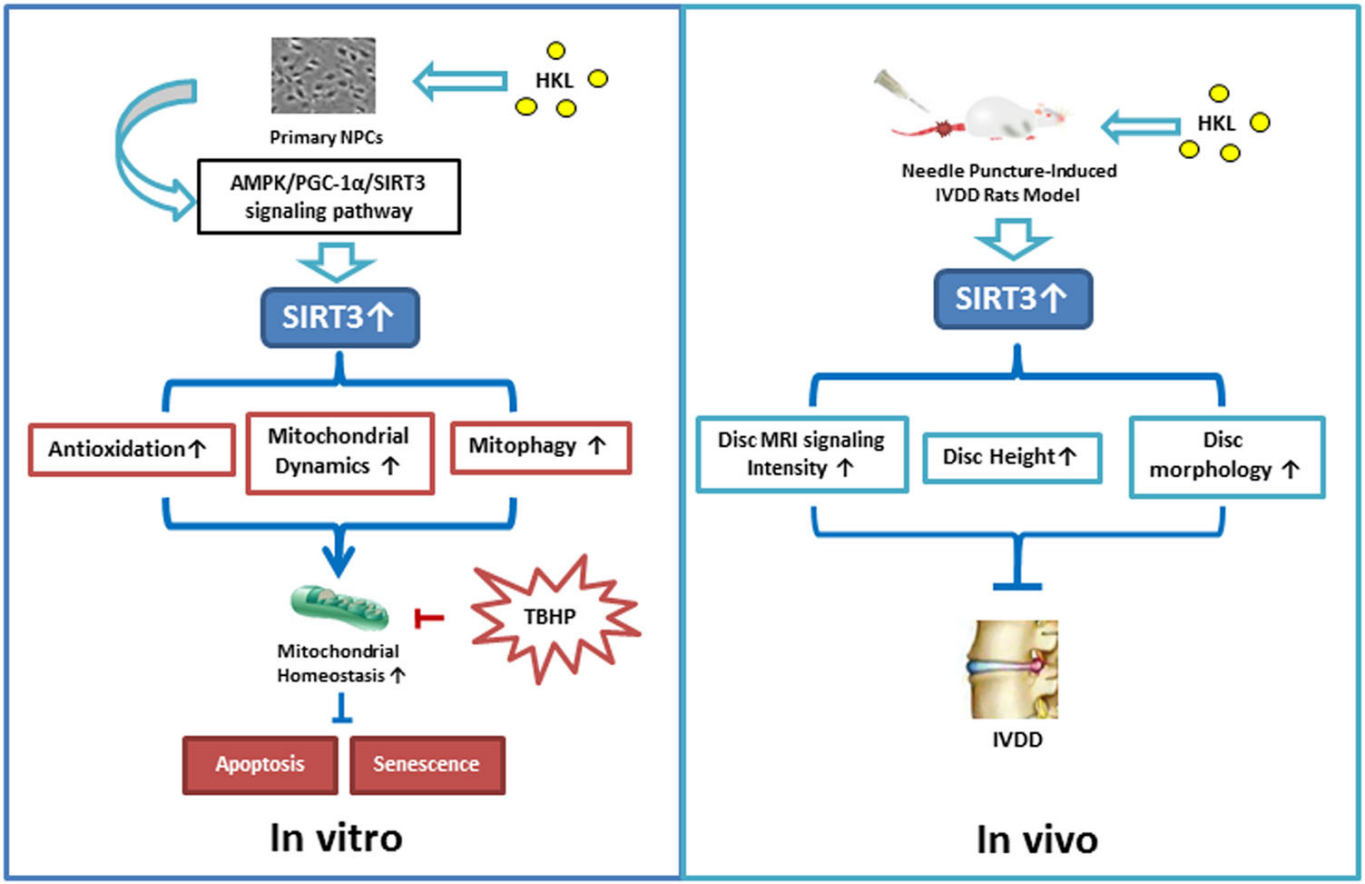
Schematic of protective effects of HKL on IVDD. In vitro, HKL activates SIRT3 via the AMPK-PGC-1α signaling pathway to facilitate anti-oxidation, mitochondrial dynamics and mitophagy to prevent mitochondrial dysfunction. By maintaining mitochondrial homeostasis under oxidative stress, HKL downregulates the expression of apoptosis- and senescence-related phenotypes in TBHP-treated NPCs. In vivo, HKL administration promoted the development of IVDD in the rat model

In this study, as shown in Fig. 8, we found differences in SIRT3 expression during the development of IVDD, determined the effect of SIRT3 deficiency on NPCs and explored the pharmacological properties of HKL as a treatment for IVDD. Briefly, we discovered that the SIRT3 level showed a significant decreasing trend with the development of IVDD. In contrast, when treated with different concentrations of TBHP, a well-recognized oxidative stress inducer, SIRT3 expression substantially increased in a dose-dependent manner compared with the expression levels of SIRT3 in the untreated control group. To confirm the role of SIRT3 in senescence, apoptosis and mitochondrial homeostasis in in vitro experiments, we showed that SIRT3 knockdown aggravated apoptosis, senescence and mitochondrial dysfunction, whereas SIRT3 overexpression exerted opposing effects in the NPCs treated with TBHP. In addition, our data show that HKL, an activator of SIRT3, is capable of protecting NPCs from senescence and apoptosis induced by TBHP in vitro. To explore the mechanism of HKL on mitochondrial homeostasis, we demonstrated that HKL was able to enhance the capacity of anti-oxidation, mitochondrial dynamics and mitophagy in NPCs. For the in vivo experiments, our study showed that HKL treatment attenuated puncture-induced rat disc degeneration, with higher levels of SIRT3 in the rat NP. To the best of our knowledge, this is the first study to describe the features of SIRT3 and the role of SIRT3 in IVDD and to evaluate HKL, an activator of SIRT3, for blocking intervertebral disc degeneration.

IVDD appears to be closely associated with SIRT3 under conditions of oxidative stress in degenerative IVD^24,25^. However, the relationship between SIRT3 and IVDD has not been previously identified. In the present study, we discovered that the SIRT3 expression level decreased with the development of IVDD, whereas oxidative stress induced by TBHP upregulated the expression of SIRT3. These findings suggest that the expression of SIRT3 decreases with IVDD and increases under conditions oxidative stress.

Consistent with the findings of previous studies by Kawamura et al.^26^ and Tseng et al.^27^, we found that oxidative stress could upregulate the level of SIRT3. However, our research seems to contradict several other studies that have supported the idea that the expression of SIRT3 can be reduced by oxidative stress^28^ and thus indicated SIRT3 may respond differently in different types of cells. Moreover, SIRT3 is not the only protein that responds to mitochondrial impairment; one study reported that SIRT4 and SIRT5 are also involved in modulating mitochondria-related activities. Therefore, it may be possible that different cells may activate different types of protein to combat mitochondrial injury.

It is widely known that oxidative stress may cause mitochondrial dysfunction, thereby resulting in apoptosis and senescence^29,30^. SIRT3 plays an important role in maintaining mitochondrial homeostasis through ameliorating the damage of oxidative stress^27^. Our study demonstrated that SIRT3 knockdown aggravated apoptosis, senescence and mitochondrial dysfunction in NPCs treated with TBHP but not in untreated NPCs, indicating that SIRT3 is able to enhance the resistance to oxidative stress in NPCs.

Mitochondrial dysfunction caused by oxidative stress leads to apoptosis and senescence in NPCs^31^, implying that SIRT3 could be a novel therapeutic target in the pathomechanism of IVDD. HKL, an activator of SIRT3, is reported to attenuate cardiac hypertrophy and kidney injury and kill cancer cells by activating SIRT3;^17^ however, it has not been applied for the treatment of IVDD. In the present study, the phenotypes of apoptosis and senescence were all suppressed by HKL by activating SIRT3 in NPCs in response to oxidative stress. These data show that HKL possesses the capacity for anti-apoptosis and anti-senescence via enhancement SIRT3 in NPCs.

According to the capacity of HKL for anti-apoptosis and anti-senescence, we further explored the mechanism of action of HKL on mitochondrial dysfunction via SIRT3 activation.

As one of the major components representing oxidative stress, O_2_·^−^ was decreased with HKL treatment in TBHP-treated NPCs; this effect was partially abolished by SIRT3 knockdown. Moreover, the MDA assay results indicated that HKL could alleviate the degree of oxidative stress via SIRT3 activation. SOD is able to convert superoxide to hydrogen peroxide followed by subsequent transformation of hydrogen peroxide into H_2_O and O_2_. We found that HKL improved SOD activity in a SIRT3-dependent manner. Therefore, we suggest that HKL is able to rescue the impaired anti-oxidation induced by TBHP via SIRT3 activation.

Mitochondria, which are highly motile and plastic organelles, constantly undergo fusion and fission, which alter the morphology of mitochondria^29^. In-time mitochondrial fusion and fission are both significant for maintaining mitochondrial quality. In our study, TBHP enhanced mitochondrial fission events, whereas it impaired fusion events. Treatment with HKL rescued the loss of Mfn2 levels and further enhanced Drp1 and Fis1 levels via SIRT3 activation, suggesting that HKL promotes mitochondrial dynamics by enhancing SIRT3.

Defective mitochondria that cannot be removed in time may be the main source of excessive ROS^32^. Mitophagy, a special type of autophagy, is capable of clearing impaired mitochondria segregated by mitochondrial fission and thus plays a crucial role in maintaining mitochondrial homeostasis^33^. In the current study, we found that HKL not only increased the levels of two mitophagic markers, Bnip3 and Bnip3L, but also promoted lipidation of the marker of autophagic vacuoles, LC3. HKL-treated NPCs showed consistently more colocalization of LC3 and Bnip3L and more autophagic vesicles that contained mitochondria than untreated NPCs. These effects of HKL on mitophagy could be abolished by SIRT3 knockdown, indicating that HKL is able to promote mitophagy in NPCs by enhancing SIRT3.

To investigate the mechanism of HKL-induced SIRT3 enhancement, we examined the activation of two major sensors of energy molecules, AMP-dependent protein kinase (AMPK) and peroxisome proliferator-activated receptor γ coactivator 1α (PGC-1α). A recent study has demonstrated that HKL is able to inhibit breast cancer in an AMPK-dependent manner^34^. PGC-1α, as a powerful transcription factor involved in the regulation of mitochondrial physiological activities, is reported to be enhanced by AMPK and promote the expression of SIRT3^28^. As expected, our study found that HKL promoted SIRT3 activation by triggering AMPK phosphorylation and subsequently upregulating the PGC-1α level.

Based on the beneficial effects of HKL in NPCs, we treated rats with HKL to evaluate its therapeutic value in vivo. Our study showed that HKL ameliorated IVDD in rats and enhanced SIRT3 expression in IVDs, indicating that HKL suppressed the development of IVDD by promoting the SIRT3 level.

In conclusion, our research indicated that there are differences in SIRT3 expression in human NPCs in vivo and TBHP-treated rat NPCs in vitro. Moreover, SIRT3 protected NPCs against apoptosis, senescence and mitochondrial dysfunction induced by oxidative stress. HKL administration rescued oxidative stress-induced apoptosis and senescence and ameliorated mitochondrial dysfunction by enhancing mitochondrial anti-oxidation, mitochondrial dynamics and mitophagy through the AMPK/PGC-1α/SIRT3 signaling pathway. Moreover, HKL activated SIRT3 and blocked the development of IVDD in vivo. These findings may provide new directions in the search for novel applications of established drugs, which can be applied to prevent IVDD.

## Electronic supplementary material

Description of suppementary file 1

supplementary file 1

## References

[1] Deyo RA, Mirza SK (2016). CLINICAL PRACTICE. Herniated lumbar intervertebral disk. N. Engl. J. Med..

[2] Vergroesen PP (2015). Mechanics and biology in intervertebral disc degeneration: a vicious circle. Osteoarthr. Cartil..

[3] Fontana G, See E, Pandit A (2015). Current trends in biologics delivery to restore intervertebral disc anabolism. Adv. Drug Deliv. Rev..

[4] Dimozi A, Mavrogonatou E, Sklirou A, Kletsas D (2015). Oxidative stress inhibits the proliferation, induces premature senescence and promotes a catabolic phenotype in human nucleus pulposus intervertebral disc cells. Eur. Cell Mater..

[5] Chen D (2016). Metformin protects against apoptosis and senescence in nucleus pulposus cells and ameliorates disc degeneration in vivo. Cell Death Dis..

[6] Zhao CQ, Zhang YH, Jiang SD, Jiang LS, Dai LY (2010). Both endoplasmic reticulum and mitochondria are involved in disc cell apoptosis and intervertebral disc degeneration in rats. Age.

[7] Blanco FJ, Rego I, Ruiz-Romero C (2011). The role of mitochondria in osteoarthritis. Nat. Rev. Rheumatol..

[8] Osellame LD, Duchen MR (2014). Quality control gone wrong: mitochondria, lysosomal storage disorders and neurodegeneration. Br. J. Pharmacol..

[9] Zhou Y (2017). Sirt3 deficiency increased the vulnerability of pancreatic beta cells to oxidative stress-induced dysfunction. Antioxid. Redox Signal..

[10] Wei T (2017). Sirtuin 3 deficiency accelerates hypertensive cardiac remodeling by impairing angiogenesis. J. Am. Heart Assoc..

[11] Sosulski ML, Gongora R, Feghali-Bostwick C, Lasky JA, Sanchez CG (2017). Sirtuin 3 deregulation promotes pulmonary fibrosis. J. Gerontol. A Biol. Sci. Med. Sci..

[12] Shi H (2017). Sirt3 protects dopaminergic neurons from mitochondrial oxidative stress. Hum. Mol. Genet..

[13] Salvatori I, Valle C, Ferri A, Carri MT (2017). SIRT3 and mitochondrial metabolism in neurodegenerative diseases. Neurochem. Int..

[14] Dikalova AE (2017). Sirt3 impairment and SOD2 hyperacetylation in vascular oxidative stress and hypertension. Circ. Res..

[15] Woodbury A, Yu SP, Wei L, García P (2013). Neuro-modulating effects of honokiol: a review. Front. Neurol..

[16] Fried LE, Arbiser JL (2009). Honokiol, a multifunctional antiangiogenic and antitumor agent. Antioxid. Redox Signal..

[17] Pillai VB (2015). Honokiol blocks and reverses cardiac hypertrophy in mice by activating mitochondrial Sirt3. Nat. Commun..

[18] General Assembly of the World Medical Association. (2014). World Medical Association Declaration of Helsinki: ethical principles for medical research involving human subjects. J. Am. Coll. Dent..

[19] Pfirrmann CW, Metzdorf A, Zanetti M, Hodler J, Boos N (2001). Magnetic resonance classification of lumbar intervertebral disc degeneration. Spine.

[20] Xu D (2017). Hydrogen sulfide protects against endoplasmic reticulum stress and mitochondrial injury in nucleus pulposus cells and ameliorates intervertebral disc degeneration. Pharmacol. Res..

[21] Zeng Z (2016). Polydatin ameliorates injury to the small intestine induced by hemorrhagic shock via SIRT3 activation-mediated mitochondrial protection. Expert. Opin. Ther. Targets.

[22] Han B (2008). A simple disc degeneration model induced by percutaneous needle puncture in the rat tail. Spine.

[23] Zemirli N, Morel E, Molino D (2018). Mitochondrial dynamics in basal and stressful conditions. Int. J. Mol. Sci..

[24] Zhang J (2016). Are sirtuins markers of ovarian aging?. Gene.

[25] Wang F, Cai F, Shi R, Wang XH, Wu XT (2016). Aging and age related stresses: a senescence mechanism of intervertebral disc degeneration. Osteoarthr. Cartil..

[26] Kawamura Y (2010). Sirt3 protects in vitro-fertilized mouse preimplantation embryos against oxidative stress-induced p53-mediated developmental arrest. J. Clin. Invest..

[27] Tseng AH, Shieh SS, Wang DL (2013). SIRT3 deacetylates FOXO3 to protect mitochondria against oxidative damage. Free Radic. Biol. Med..

[28] Zhou X (2014). Resveratrol regulates mitochondrial reactive oxygen species homeostasis through Sirt3 signaling pathway in human vascular endothelial cells. Cell Death Dis..

[29] Kim HS (2010). SIRT3 is a mitochondrial localized tumor suppressor required for maintenance of mitochondrial integrity and metabolism during stress. Cancer Cell..

[30] Ballinger SW (2000). Hydrogen peroxide- and peroxynitrite-induced mitochondrial DNA damage and dysfunction in vascular endothelial and smooth muscle cells. Circ. Res..

[31] Shen J (2017). IL-1β induces apoptosis and autophagy via mitochondria pathway in human degenerative nucleus pulposus cells. Sci. Rep..

[32] Dagda RK, Kulich SM, Tandon A, Park D, Chu CT (2009). Loss of PINK1 function promotes mitophagy through effects on oxidative stress and mitochondrial fission. J. Biol. Chem..

[33] Frank M (2012). Mitophagy is triggered by mild oxidative stress in a mitochondrial fission dependent manner. Biochim. Biophys. Acta Mol. Cell Res..

[34] Sengupta S (2017). Activation of tumor suppressor LKB1 by honokiol abrogates cancer stem-like phenotype in breast cancer via inhibition of oncogenic Stat3. Oncogene.

