# Supplementary material for: Small molecule natural compound agonist of SIRT3 as a therapeutic target for the treatment of intervertebral disc degeneration
Source: Exp Mol Med. 2018 Nov 12;50(11):146. doi: 10.1038/s12276-018-0173-3 (PMC6232087; doi:10.1038/s12276-018-0173-3)
Supplement: Supplementary file 1 — Description of suppementary file 1 [file 12276_2018_173_MOESM1_ESM.docx]

Supplementary file 1 Characteristic phenotypes collagen II and MMP-3 in NPCs treated with TBHP. Scale bar 50 μM. (A, B) The effect of TBHP on expression of collagen II and MMP-3 in NPCs. All experiments were performed as mean ± SE SD of three times in duplicates. *P < 0.05, **P < 0.01.
