# Supplementary figures and images for: Small molecule natural compound agonist of SIRT3 as a therapeutic target for the treatment of intervertebral disc degeneration
Source: Exp Mol Med. 2018 Nov 12;50(11):146. doi: 10.1038/s12276-018-0173-3 (PMC6232087; doi:10.1038/s12276-018-0173-3)

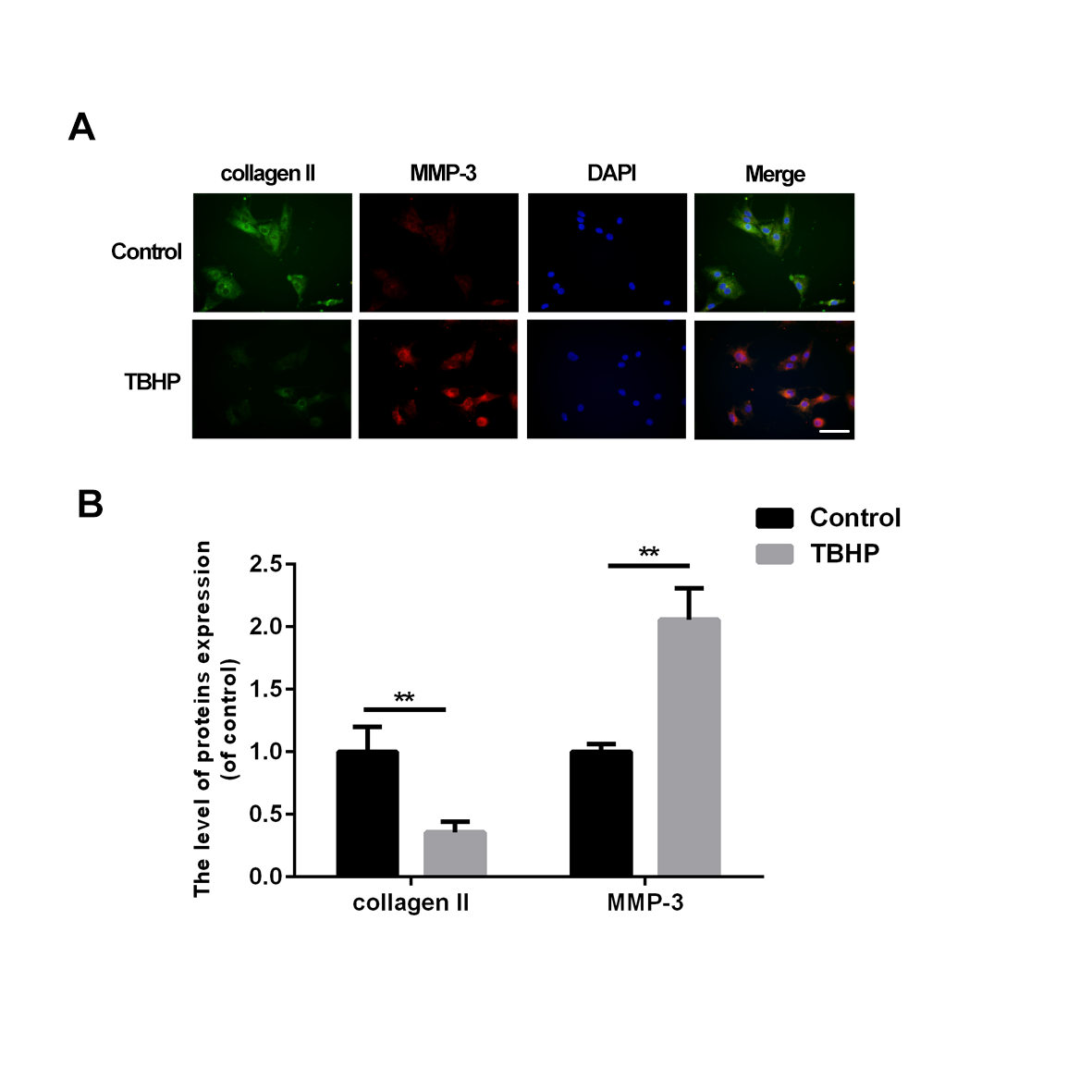

Supplement: Supplementary file 2 — supplementary file 1 [file 12276_2018_173_MOESM2_ESM.tif]
